# In silico design and ADMET evaluation of new inhibitors for PIM1 kinase using QSAR studies, molecular docking, and molecular dynamic simulation

**DOI:** 10.1016/j.heliyon.2024.e38309

**Published:** 2024-09-24

**Authors:** Fereshteh Golestanifar, Zahra Garkani-Nejad

**Affiliations:** a Chemistry Department, Faculty of Science, Shahid Bahonar University of Kerman, Kerman, Iran; b Young Researchers Society, Shahid Bahonar University of Kerman, Kerman, Iran

**Keywords:** PIM1 kinase, QSAR, Molecular docking, MD simulation, ADMET evaluation

## Abstract

The proviral Integration site of Moloney (PIM) kinase is highly expressed in various diseases, including cancer, making the development of selective inhibitors for this protein important. A series of PIM1 inhibitors, triazolo [4, 3-b] pyridazin-3-yl-quinoline derivatives, have been studied to design new inhibitors. The activity and structural features of these derivatives were investigated to understand their interactions with PIM1 using molecular docking, molecular dynamic simulation, and QSAR techniques.

In a study of 30 compounds using the structure-activity technique and the MLR method, a linear model with R^2^_train_ = 0.91 and R^2^_test_ = 0.96 was obtained. The model utilized descriptors such as RDF080v, RDF105v, RDF135v, Mor03v, and H046 to express the structural characteristics of the inhibitors. To enhance the model, the SVR non-linear method with the RBF function was also used, resulting in an improved model with R^2^_train_ = 0.98 and R^2^_test_ = 0.98.

Furthermore, the molecular docking technique was employed to investigate the interaction of compounds with high (compound 25) and low (compound 13) inhibitory activity. It was observed that the rings with nitrogen atoms interacted with the protein. The molecular binding results indicate that groups such as OMe and rings with Nitrogen can enhance the inhibitory activity of the compounds. Additionally, oxygen and nitrogen atoms contribute to an increased number of hydrogen bonds, thereby increasing the inhibitory activity of the compounds.

Additionally, the stability and bonding modes of active and inactive compounds were studied using molecular dynamic simulation. Based on the results, four new inhibitors were designed, demonstrating better inhibition efficiency with the PIM1 kinase compared to the reference compounds. Moreover, the designed compounds underwent evaluation for ADMET, yielding promising results.

## Graphical abstract

**Figure:**
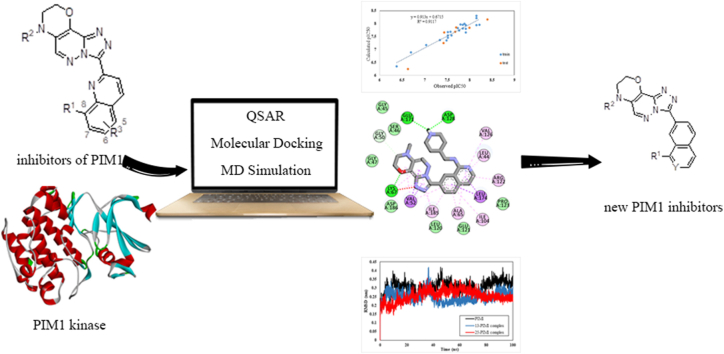

## 1 Introduction

One member of the serine/threonine kinase family is PIM kinase, which plays an important role in activating and regulating cell proliferation, cell cycle regulation, and inhibiting apoptosis [1,2]. This family consists of three isoforms: PIM1, PIM2, and PIM3, which share a high level of sequence homology and exhibit some functional redundancy [3]. PIM1 is expressed at higher levels in hematopoietic cells, advanced lymphoid tissue of T-cell lymphoma, myeloid leukemia (AML), B-cell non-Hodgkin's lymphoma, and solid tumors such as prostate cancer. On the other hand, PIM2 is more highly expressed in brain, leukemia, and lymphoid cells, while PIM3 shows higher levels in kidney, breast, and brain cells. Dysfunction or excessive expression of these proteins can indicate various blood malignancies and solid tumors [[4], [5], [6]]. PIM1 kinase regulates cytokine signaling by controlling multiple transcription factors and various signaling pathways [7].

The cyclin-dependent kinase family is widely studied as it is a major drug target for modulating various biological processes. PIM1, a distinct serine/threonine kinase fold, consists of two contiguous domains connected by a hinge region. The N-terminal domain is primarily composed of β-sheets, while the C-terminal domain is primarily composed of β-helices. The ATP-binding pocket is formed by a groove at the interface between the N- and C-terminal domains [7]. PIM1 kinase has a binding pocket for ATP in the hinge region, which binds to inhibitors from this region.

Several series of inhibitors, including Imidazopyridazines [8], 1, 2, 3- triazolo [4, 5- b] pyridines [9], thiazolidine- 2, 4-dione [10], 1,2,4,5-Tetrazine [11], pyrazolo-pyrimidine [12] and thiazolidine [13] analogs or derivatives, that include several heterocyclic rings such as pyrrolo, pyrimidine, thiazolidine, benzofuran, indole, triazole, oxadiazole, and quinoline derivatives, were developed as PIM kinase inhibitors at the laboratory level. All these derivatives have a specific ring or functional groups which are associated with the PIM kinase inhibitory activity. For example, in pyrrolo and pyrazolo derivatives, the positions from C-6 to C-9 are important for describing the inhibitory activity against the PIM1 kinase. The presence of a halogen atom at the C-6 position can increase the potential inhibitory activity. Substituting the pyrrolo[2,3-a] carbazole scaffold at C-6 or C-9 positions with methoxycarbonyl, bromine, trifluoromethyl, or ethyl groups has been found to produce compounds with improved inhibitory potencies toward PIM1. Additionally, the synthesis of pyrrolo[2,3-g] indazole derivatives represents a significant step in the development of emerging PIM kinase inhibitors. Also, the anti-proliferative activity of the quinoline derivatives is dependent on the secondary amine linked with quinoline and a pyridine ring [14]. Virtual screening can predict the efficacy, stability, and pharmacokinetic properties of drugs prior to synthesis, thereby reducing the time, cost, and effort needed for novel medication discovery [15].

Imidazo [1, 2-b] pyridazine compounds like SGI-1776 are known as PIM1 kinase inhibitors [16]. Numerous studies have explored the anticancer activity of quinoline derivatives using experimental and QSAR methods [[17], [18], [19]].

The QSAR model development can facilitate the discovery and optimization of PIM1 Kinase inhibitors by analyzing their structural features. This is achieved by interpreting the interaction mechanism between the ligand and protein through molecular docking and molecular dynamics simulation. The combined use of QSAR, molecular docking, and molecular dynamics simulation provides detailed information about the interaction mechanism, which can be utilized in the design of new drug compounds [20]. Also, ADMET is a process to evaluate the pharmacological properties of new compounds, involving Absorption, Distribution, Metabolism, Excretion, and Toxicity.

A series of Imidazo[1,2-b] pyridazine quinoline derivatives have been reported in the literature. Their inhibitory activities against PIM1, PIM2, and PIM3 kinases were measured [5], prompted further study of these compounds using various computational methods.

This paper investigates the detailed binding modes and inhibition of imidazo[1,2-b] pyridazine quinoline derivatives against the PIM1 kinase through QSAR modeling, molecular docking, and MD simulations. The 3D structures of inhibitors were optimized using quantum chemistry methods, and then docked into the PIM1 kinase to generate the most stable structural state. The detailed interactions of these compounds were elucidated from the results of the molecular docking and MD simulations. In the process of QSAR modeling, the optimized structures of the inhibitors were utilized to calculate structural descriptors and generate a model. According to the results of the QSAR modeling and the ligand – PIM1 interactions obtained using molecular docking, four new inhibitors with improved inhibition efficiency were successfully designed. The interactions of newly designed compounds with the receptor were also investigated using molecular docking and MD simulation. Furthermore, the designed compounds were also subjected to ADMET evaluation, which yielded promising outcomes.

## 2 Computational methodology

### 2.1 Selecting of data set

A batch of 30 triazolo [4, 3-b] pyridazin-3-yl-quinoline derivatives was used in this study as inhibitors of PIM1 kinase. The inhibitory activities of these derivatives on PIM2 kinase and PIM3 kinase were also investigated alongside PIM1 kinase [5]. The experimental results indicate that these compounds have a suitable inhibition profile for PIM1 and PIM3, but poor inhibition for PIM2. Analysis of the PIM kinases reveal that PIM2 and PIM3 are 55 % and 71 % similar to PIM1, respectively. The inhibition activity was measured using the IC50 parameter, which was converted to pIC50 (-log IC50 in Molar) for this project. The structure of the studied compounds, along with their IC50 and pIC50 values, can be found in Table 1.

**Table 1:**
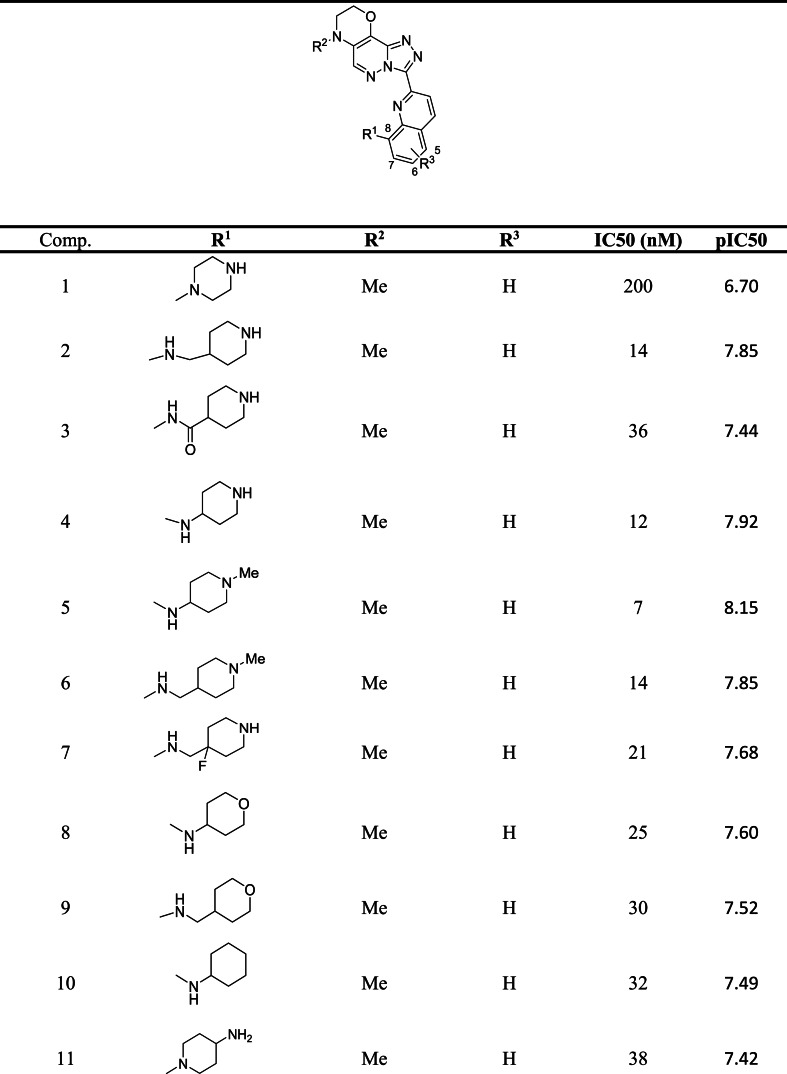

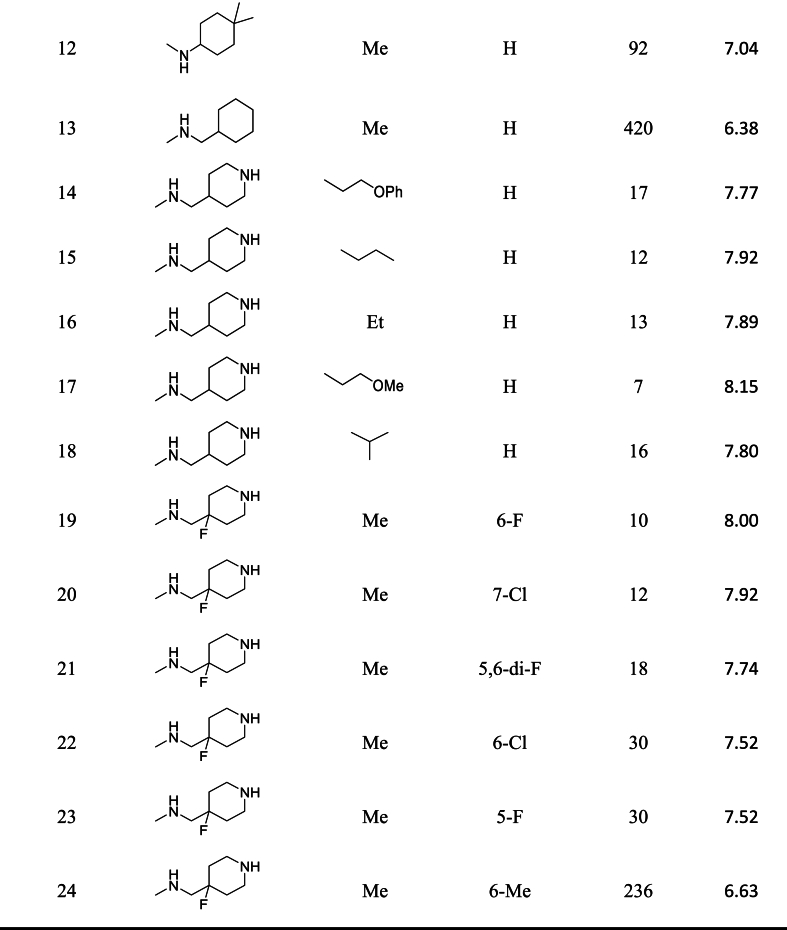

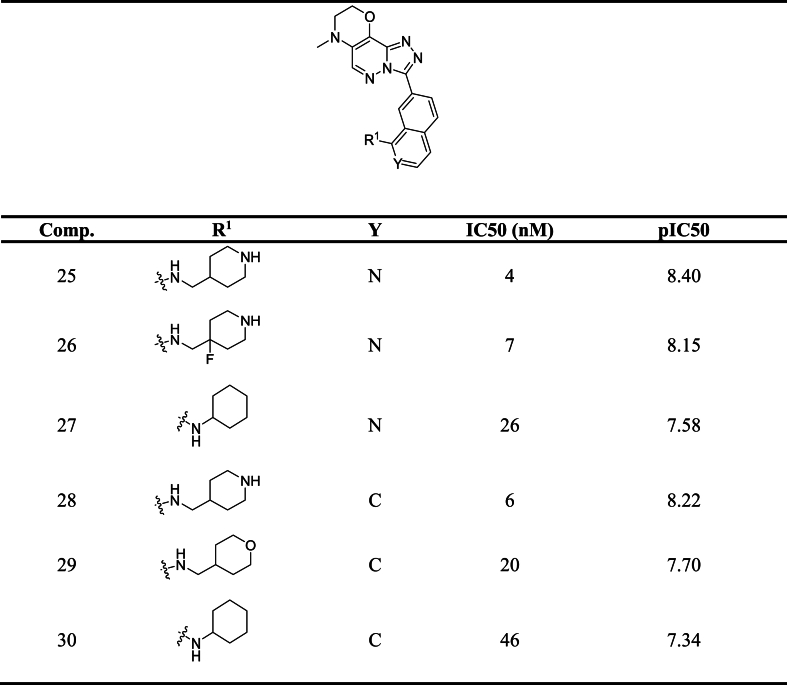
Structure of PIM1 kinase inhibitors with reported IC50 and pIC50 values.

### 2.2 Ligand preparation and optimization

The structures of PIM1 Inhibitors were drawn using Gauss View (version 5.0.8) software [21]. Subsequently, the optimization of these compounds was carried out using Gaussian 09 software [22,23] with high-precision quantum chemistry methods. In this study, all the compound structures were optimized employing DFT using the B3LYP [24,25] method and 6-31G∗ basis set [26]. The structure with the minimum energy of each compound was used as the input file to calculate various descriptors for QSAR analysis, molecular dynamic simulation, and molecular docking.

### 2.3 QSAR

#### 2.3.1 Molecular descriptor calculation

Some electronic descriptors were calculated using Gaussian 09 software. Then, the optimized structures of the molecules were imported into the Hyperchem software (Version 8.0) [27] to calculate physicochemical parameters such as molecular polarizability (MP), hydrophobicity (log P), molecular volume (V), and surface area (S). Subsequently, the Hyperchem files were imported into Dragon 3.0 software [28] to compute structural, topological, geometric, radial, and electrostatic distribution functions. Finally, the 620 calculated descriptors were used as input for statistical analysis in IBM SPSS Statistics 25.0 [29] and IBM SPSS Modeler 18.0 [30] softwares.

#### 2.3.2 Statistical analysis

After calculating all descriptors, they were used as input for a QSAR study on the pIC50 values of the ligands until a valid model was obtained. QSAR studies aim to obtain models with excellent predictive ability. The data for the QSAR study were divided into two categories: training and test series. 77 % of the compounds (23 compounds) were selected for the training set, while 23 % of the compounds (7 compounds) were assigned to the test set [31]. The test set samples were chosen to contain both active and inactive species. The pIC50 values were used as dependent variables, and stepwise multiple linear regression (MLR) and support vector regression (SVR) were employed to build quantitative models.

The R^2^ (coefficient of determination), standard error of estimation (SEE), and F-test are used to assess the suitability of the models obtained. Y randomization was employed to demonstrate the robustness and predictability of the model.

### 2.4 Molecular docking

**Fig. 1:**
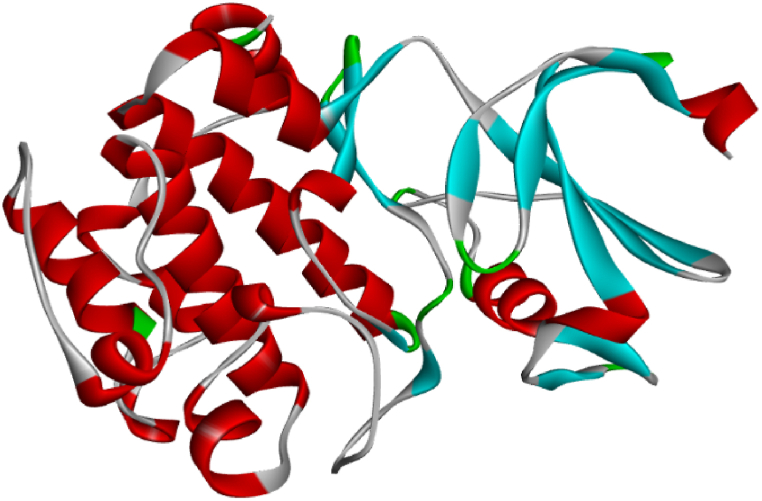
Structure of PIM1 kinase (PDB code: 4ty1).

Molecular docking is a method used to analyze the interactions between molecules and determine the optimal positioning of a molecule within a complex. In this study, AutoDock software (version 4.2) [32]was utilized to investigate the molecular docking of triazolo [4, 3-b] pyridazin-3-yl-quinoline derivatives on the binding site of PIM1 kinase, using the crystal structure of PIM1 kinase with PDB code 4ty1 obtained from the protein data bank (Fig. 1). The protein and ligand structures were docked using Auto Dock Tools. Upon importing the protein into the software environment, water molecules were removed from the structure, and hydrogens and kolleman charges were added. The Auto Grid program was employed to calculate grid parameters, with a grid size set to 126 × 126 × 126 points and a grid spacing of 0.375 Å. The Lamarckian genetic algorithm was utilized for structural search [33] with 100 runs and distinct structural clusters formed using an RMSD tolerance of 2.0 Å. During the docking process, the PIM1 kinase was kept rigid while the ligands were allowed to be completely flexible. The interactions between the ligands and PIM1 kinase were analyzed using Discovery Studio visualizer v16.1.0.15350 [34]. Additionally, the study included pharmacophore mapping of the best ligands, which was carried out using Molegro Virtual Docker 6.0 software.

### 2.5 Docking validation

The accuracy of the docking results was confirmed by taking the co-crystallized ligand from the protein (PDB Id: 4ty1) and re-docking it into its original position. The lowest energy pose from the re-docking was compared to the co-crystallized ligand, and the root mean square deviation (RMSD) between the two superimposed ligands was calculated. To ensure the reliability of the docking process, the RMSD should be less than 2 Å [35].

### 2.6 Molecular dynamics simulation

Molecular dynamics simulations were conducted to study the stable binding of ligands to the PIM1 kinase. The simulations provided detailed pathways and information about the stability and interaction of inhibitors with the PIM1 kinase [36]. The simulations were performed using the Gromacs (2018.1) software package [37,38] and the GROMOS96 43a1 force field in cubic boxes [39]. The crystal structure with code 4ty1 from the protein database site (http://www.rcsb.org) was selected at a resolution of 2.70 Å for this study. The appropriate molecular topology file (.itp) was obtained using the PRODRG web server (https://prodrg2.dyndns.org) [40]. Following this, water molecules were added using a simple charge (SPC216) model and the system was neutralized by adding sodium ions (Na+) and chloride ions (Cl-). Energy minimization was performed, followed by equilibration in two phases. The first phase involved "isothermal-isochoric" equilibration under an NVT ensemble, during which the system reached a temperature of 300 K. The next phase involved pressure equilibration, also known as "isothermal-isostatic", under an NPT ensemble, where the pressure of the system stabilized at 1 bar. Subsequently, a 100 ns production MD Simulation run was conducted, maintaining the temperature at 300 K and the pressure at 1 bar.

## 3 Results and discussion

### 3.1 QSAR study

Based on the descriptors obtained from HyperChem and Dragon software, a mathematical linear model was developed to quantitatively predict the physicochemical effects of triazolo[4,3-b] pyridazin-3-yl-quinoline derivatives on PIM1 activity using multiple linear regression. Multiple linear regression is a statistical technique used to predict the value of one variable based on two or more variables. The variable to be predicted is defined as the dependent variable, while the variables used to predict its value are defined as the independent variables. Using SPSS 25 software and the multiple linear regression (MLR) method, linear modeling was conducted to predict the inhibitory activity of the training set, resulting in the following model.

(1) pIC50=(5.038±0.437)+(0.288±0.055)×RDF105v–(1.085±0.14)×RDF135v–(0.139±0.013)×H046–(0.425±0.115)×Mor03v+(0.046±0.014)×RDF080v

In Equation (1), pIC50 represents inhibitory activity, while RDF v, Mor03v, and H046 are descriptors used in the model. The statistical parameters for the MLR modeling based on the training and test sets are presented in Table 2.

**Table 2:** MLR modeling results for PIM1.

set	n	R^2^	Std. Error	F
train	23	0.91	0.15	34.94
test	7	0.96	0.27	4.76

Based on Table 2, the coefficient of determination (R^2^ = 0.91) indicates a high level of predictive capability, along with a low standard error value of 0.15 and a high F value of 34.94 based on the training set. This suggests that the developed model has strong predictive capabilities. The model also demonstrates good fitting capability for the entire data set. Table 3 presents the experimental values of pIC50 and the predicted values using equation (1) for all compounds.

**Table 3:** Values of molecular descriptors, experimental and predicted inhibitory activity of target compounds.

compound	RDE080v	RDF105v	RDF135v	H046	Mor03v	pIC50 exp.	pIC50 cal. (MLR)	pIC50 cal. (SVR)
training set								
**1**	6.82	1.86	0.12	0	−2.41	6.70	6.78	6.85
**2**	12.26	2.10	0.03	0	−3.80	7.85	7.79	7.70
**4**	6.93	3.94	0.12	0	−3.39	7.92	7.80	7.77
**5**	11.15	3.36	0.11	0	−3.26	8.15	7.79	8.00
**6**	11.88	3.19	0.09	0	−2.98	7.85	7.67	7.70
**8**	8.21	2.76	0.11	0	−3.08	7.60	7.40	7.63
**9**	10.75	2.26	0.12	0	−3.17	7.52	7.40	7.62
**10**	9.52	3.89	0.13	6	−3.77	7.49	7.22	7.59
**12**	13.56	4.75	0.11	10	−3.63	7.04	7.06	7.19
**13**	9.52	2.48	0.12	10	−3.71	6.38	6.24	6.53
**14**	9.24	2.50	0.12	0	−3.61	7.77	7.58	7.62
**15**	15.92	3.23	0.12	3	−3.18	7.92	7.50	7.77
**16**	10.43	2.67	0.10	0	−3.84	7.89	7.81	7.74
**17**	12.13	4.11	0.25	0	−3.83	8.15	8.13	8.00
**18**	11.84	2.13	0.06	0	−3.90	7.80	7.79	7.65
**20**	6.91	2.88	0.10	0	−3.70	7.92	7.65	7.77
**21**	12.5	2.51	0.10	0	−3.39	7.74	7.67	7.62
**22**	13.31	2.25	0.47	0	−3.50	7.52	7.28	7.62
**23**	12.40	2.27	0.16	0	−3.33	7.52	7.51	7.63
**26**	14.80	2.39	0.08	0	−3.99	8.15	8.02	8.00
**27**	14.79	3.95	0.05	6	−3.71	7.58	7.54	7.59
**28**	11.91	1.86	0.01	0	−3.92	8.22	7.78	8.07
**30**	12.58	3.05	0.00	6	−3.70	7.34	7.22	7.49
test set								
**3**	8.82	2.58	0.10	0	−3.38	7.44	7.66	7.59
**7**	11.98	2.38	0.12	0	−3.25	7.68	7.69	7.62
**11**	7.59	2.36	0.12	0	−2.79	7.42	7.25	7.57
**19**	12.11	2.55	0.07	0	−3.33	8.00	7.84	7.85
**24**	13.25	3.02	1.30	3	−3.29	6.63	6.24	6.78
**25**	14.64	2.41	0.09	0	−3.94	8.40	8.17	8.25
**29**	10.96	2.61	0.02	0	−3.34	7.70	7.85	7.61

**Fig. 2:**
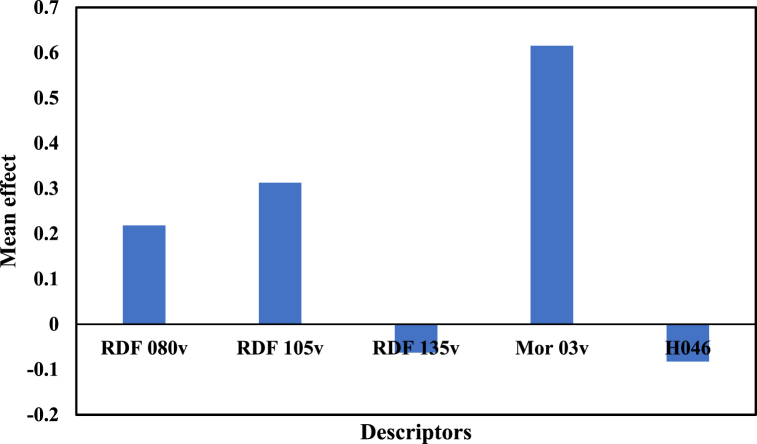
Mean effect of molecular descriptors in QSAR model.

The linear model (Eq. (1)) comprises 5 descriptors: RDF 105v, RDF 135v, H046, Mor03v, and RDF080v. The values and mean effects of these descriptors are presented in Table 3 and Fig. 2, respectively. Three parameters RDF080v, RDF105v, and RDF135v are Radial Distribution Function descriptors, defined as a radial distribution function weighted by atomic van der Waals volumes. These descriptors can transform 3D coordinates of the atoms of molecules into a structured code with a specified number of descriptors, regardless of the molecule's size. Based on the results, an increase in RDF080v and RDF105v values indicates an increasing effect on activity, while an increase in RDF 135v value shows a decreasing effect on the inhibitory activity of the compounds.

Mor03v is defined as 3D-MoRSE-signal 03 weighted by atomic van der Waals volumes. The positive sign of the regression coefficient of Mor03v indicates that an increase in the value of this descriptor leads to an increase in inhibitory activity.

The H046 descriptor, which is an atom-centered fragment, is defined as H attached to C0 (sp3) with no X attached to the next C. An increase in the value of H-046 leads to a decrease in the inhibitory activity of the compounds.

Multicollinearity is a statistical issue that arises when one input variable is highly correlated with multiple other input variables. This indicates that one input variable can be predicted based on another input variable, leading to a significant impact on the accuracy of the model's output. Therefore, it is important to assess the multicollinearity among the input parameters [41].

The presence of multicollinearity was examined by assessing the variance inflation factor (VIF). The variance inflation factor (VIF) for the descriptors was computed. It is recommended that the VIF value should be below 5 [42]. The low values (VIF<2) indicate that the measurements are precise (Table 4).

**Table 4:** Collinearity Statistics of the descriptors.

variables	VIF
RDF 080v	1.15
RDF 105v	1.36
RDF 135v	1.07
Mor 03v	1.16
H046	1.45

The descriptors used in Equation (1) were also applied to PIM2 and PIM3 kinases. Equations (2), (3)) represent the models developed for PIM2 and PIM3, respectively. The statistical parameters for these models can be found in Table 5.

(2) pIC50=4.680(±0.99)+0.013(±0.046)RDF080v–0.055(±0.16)RDF105v−0.28(±0.45)RDF135v–0.664(±0.31)Mor03v–0.038(±0.04)H046

(3) pIC50=3.424(±0.99)+0.091(±0.042)RDF080v–0.039(±0.15)RDF105v+3.004(±1.15)RDF135v–0.422(±0.28)Mor03v–0.211(±0.038)H046

**Table 5:** MLR modeling results for PIM2 and PIM3.

protein	n	R^2^	Std. Error	F
PIM2	26	0.24	0.54	1.54
PIM3	28	0.72	0.50	11.60

Support Vector Machine (SVM) is a reliable method used for classification and regression to maximize predictive accuracy without overfitting the training data. It is particularly effective for analyzing data with a large number of predictive fields. The analysis was conducted using SPSS Modeler 18.0 software, with two sets of training and testing data from the linear regression stage used as input. The kernel RBF function was employed for the calculations.

A non-linear relationship between the inhibitory activity of the compounds and the descriptors entered into the linear model was obtained. The non-linear model obtained from the SVR method showed a higher correlation coefficient than the linear model (Table 6).

**Table 6:** Statistical parameters using the SVR method.

set	n	R^2^	Std. error
train	23	0.97	0.13
test	7	0.98	0.14

To check the robustness of the MLR model, the Y randomization test was performed. The Y randomization method is a commonly employed strategy to guarantee the strength of a QSAR model [43]. In the test, the pIC50 values were randomly shuffled and a new QSAR model was created using the new values. The results of the Y randomization test are presented in Table 7. The low values of R^2^ in the Y randomization test show that there is no chance correlation in the presented MLR model.

**Table 7:** Y-Randomization evaluation results.

No. of model	R^2^	Std. Error
1	0.23	0.45
2	0.29	0.43
3	0.25	0.44
4	0.19	0.35
5	0.30	0.40

**Fig. 3:**
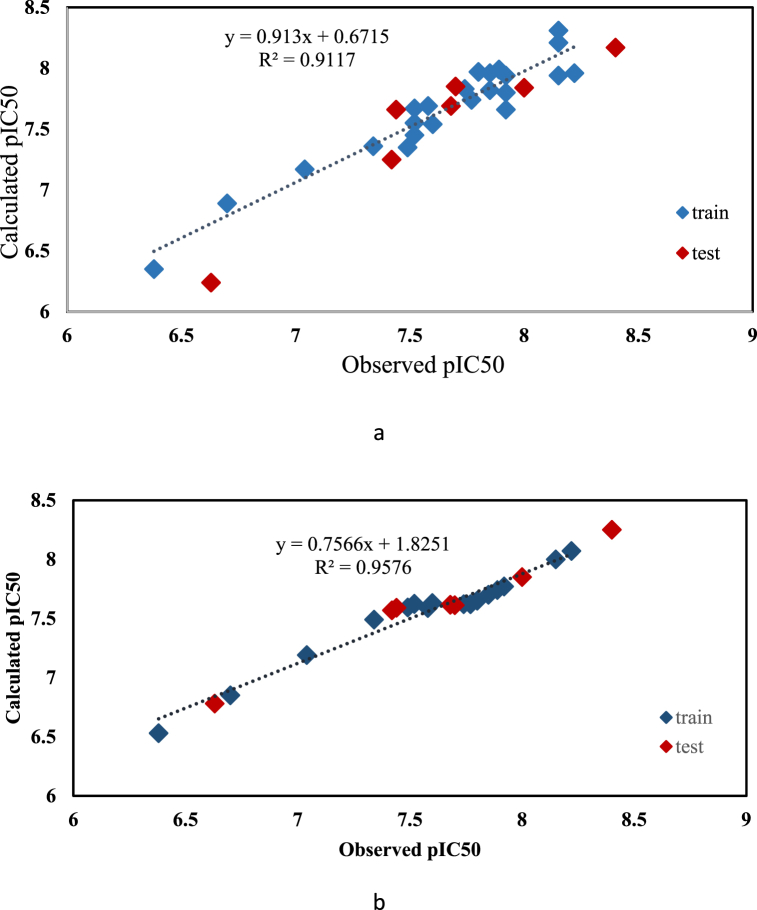
Correlation diagram of calculated values of pIC50 to experimental values for molecules of training and test sets: (a) MLR method, (b) SVR method.

The graph of predicted values against observed values is drawn for MLR and SVR models, which shows the linear correlation between predicted and observed values (Fig. 3(a and b)).

**Fig. 4:**
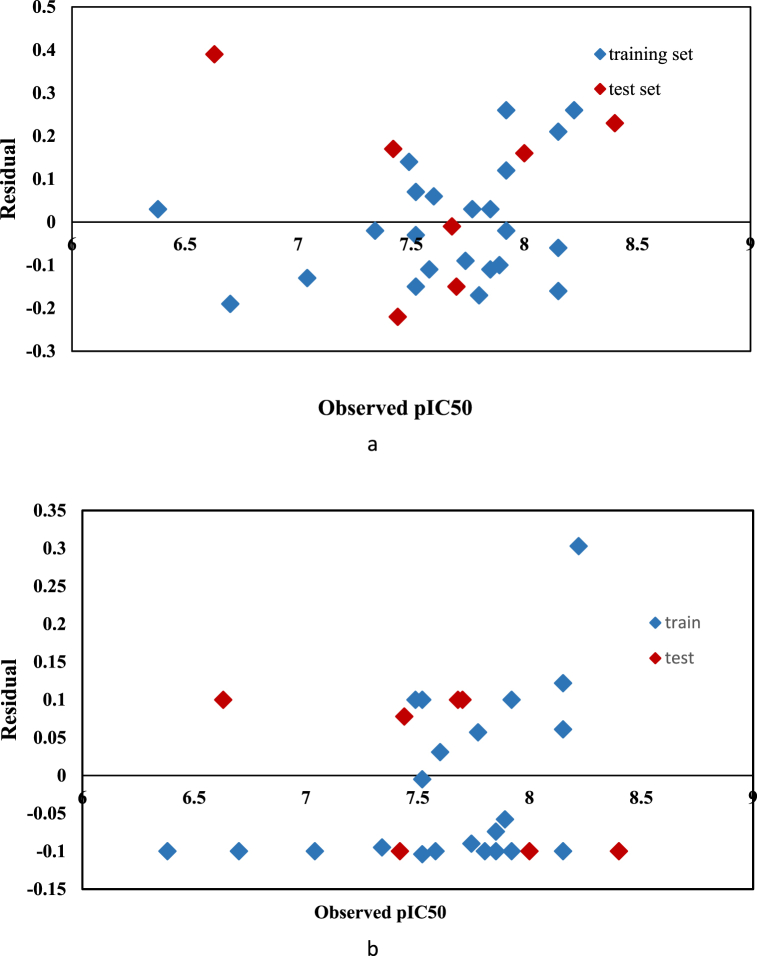
Graph of residual values relative to experimental values for training and test sets: (a) MLR method, (b) SVR method.

Fig. 3 (a, b) illustrates that the predicted pIC50 values align with the observed pIC50 values. Both the MLR and SVR methods demonstrate that the resulting models are valid and justifiable. Additionally, Fig. 4 (a, b) depicts a graph of residual values compared to experimental values. The symmetrical distribution of points around the zero axis and their balanced distribution on both sides of the line indicate the absence of systematic error in the results. Given the linear regression model's ability to effectively describe the experimental inhibition efficiency, this method can be utilized in the design of new drug compounds.

### 3.2 Molecular docking and pharmacophore mapping

Molecular docking was utilized to demonstrate the interaction mechanism between derivatives and PIM1 kinase. The identification of binding and structural cavities on the protein surface, which primarily represent active sites for protein-ligand binding, aids in locating accessible and inaccessible internal protein regions. In this study, we investigated the interaction between compounds 25 and 26, which have the lowest IC50 values and are considered active compounds, and compounds 13 and 24, which have the highest IC50 values and are considered to have low activation with PIM1. This investigation was conducted using AutoDock 4.2 software. The docking results for the best-docked conformations of these compounds are presented in Table 8. Compounds 25 and 26 exhibited binding energies of −12.12 and −12.10 kcal/mol and inhibitory constants of 1.3 and 1.3 nM, respectively, at their best states. Due to the structural similarity of these two compounds and the proximity of their IC50 values, the docking results were also very similar. Additionally, compounds with lower activity, such as 13 and 24, displayed lower binding energy and higher inhibitory constants (Table 8).

**Table 8:** Results of docking study for active and inactive compounds.

Comp.	Binding energy	Ligand efficiency	Inhib. constant	Intermol energy	IC50(nM)
25	−12.12	−0.38	1.3	−13.31	4
26	−12.12	−0.37	1.3	−13.31	7
13	−10.38	−0.32	24.7	−11.57	420
24	−11.78	−0.35	2.3	−12.98	236

**Fig. 5:**
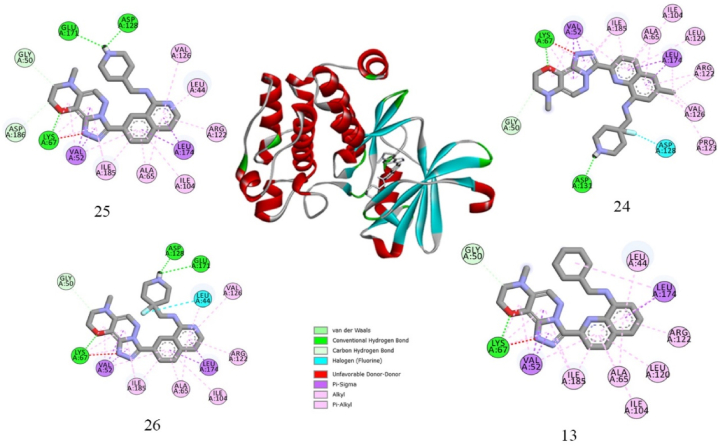
2D diagram of binding position and binding mode between PIM1 and compounds 25 and 26 with high inhibitory activity and compounds 13 and 24 with less inhibitory activity.

Compound 25, the most active compound with the lowest IC50 value, formed 4 hydrogen bonds with PIM1, including 3 conventional hydrogen bonds between the nitrogen atom of the ligand and the residues of ASP 128 and GLU171, with bond lengths of 1.70 and 2.21 Å, respectively, and between the oxygen atom of the ligand and the LYS 67 residue, with a bond length of 2.01 Å, as well as one carbon-hydrogen bond between the carbon atom and the GLY50 residue, with a bond length of 3.38 Å. Compound 26, another PIM1 inhibitor with the same IC50 as compound 25, formed four hydrogen bonds with the ASP128, GLU171, LYS67, and GLY50 residues of PIM1 at the binding site with bond lengths of 1.73, 2.31, 1.96 and 3.35 Å, respectively. Also, a halogen bond formed between the fluorine atom of this compound and the LEU44 amino acid, with a bond length of 3.35 Å. The interaction of compound 13, with the lowest inhibitory activity, was also investigated (Fig. 5). This compound formed one conventional hydrogen bond between the oxygen atom and the LYS67 residue with bond lengths of 2.08 Å and one carbon-hydrogen bond between the carbon atom and the GLY50 residue with bond lengths of 3.45 Å. Compound 24, with moderate inhibitory activity, formed 3 hydrogen bonds with the LSY67, GLY50, and ASP128 residues.

**Fig. 6:**
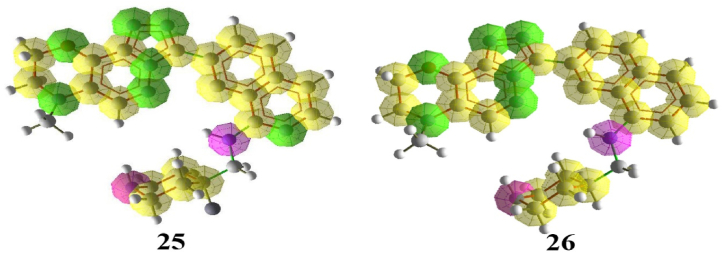
Pharmacophore Mapping of compounds 25 and 26. Here, green color-hydrogen bond acceptor, yellow color-aromatic, purple color-hydrogen bond donor.

Pharmacophore mapping was performed for compounds 25 and 26, which exhibited the highest inhibitory activity. The results (Fig. 6) showed 7 hydrogen acceptor bonds, 2 hydrogen donors, and 4 aromatic rings for compound 25, and 6 hydrogen acceptor bonds, 2 hydrogen donors, and 4 aromatic rings for compound 26. The interactions established during the docking process, Pharmacophore mapping, and the model derived from the QSAR method offer insights into the relationship between the compound's structure and inhibitory activity. The molecular binding results indicate that groups such as OMe and rings with Nitrogen can enhance the inhibitory activity of the compounds. Additionally, oxygen and nitrogen atoms contribute to an increased number of hydrogen bonds, thereby increasing the inhibitory activity of the compounds.

### 3.3 Docking validation

**Fig. 7:**
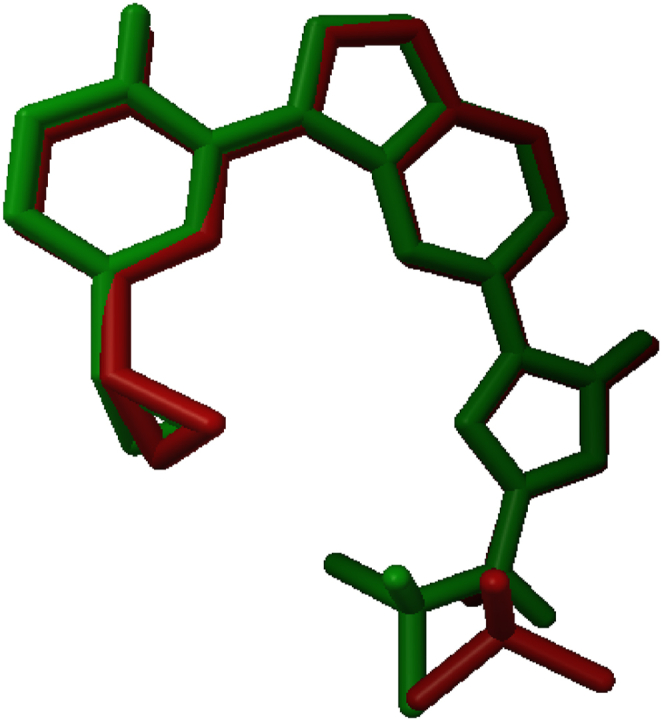
Re-docking pose with the RMSD value of 0.861 Å (Green: Original, Red: Docked).

To assess the accuracy of docking in predicting the conformation of the protein-bound ligand, re-docking of the Co-Crystallized Ligand has been performed to validate the precision of the docking process. Fig. 7 shows the comparison between the docked ligand conformation and the co-crystallized ligand conformation, with an RMSD value of 0.861 Å.

**Fig. 8:**
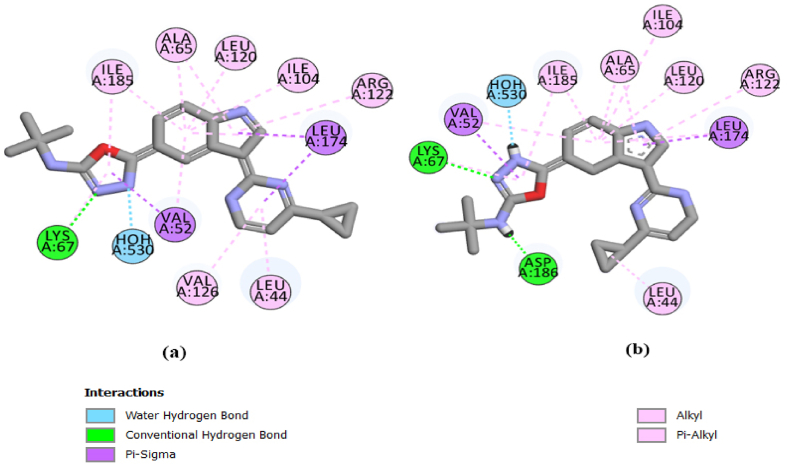
(a) 2D visualization showing interactions of original ligand. (b) 2D visualization showing interactions of the redocked ligand.

Fig. 8a, b presents a 2D visualization for inspecting the interactions between a generated docking pose and the experimental ligand conformation, confirming the interactions observed in the experimental binding mode.

### 3.4 MD simulation analysis

**Fig. 9:**
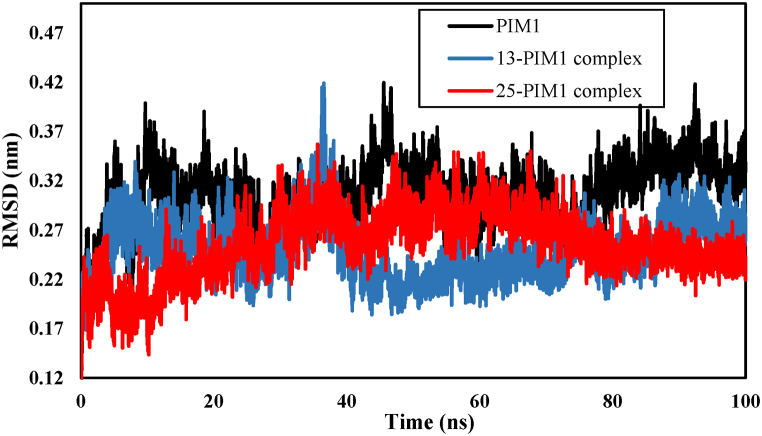
The root-mean-square-deviations (RMSD) of the backbone atoms of PIM1 kinase, complex 25- PIM1 and complex 13-PIM1.

In order to assess the binding stability and determine the active site of the PIM1 kinase when interacting with inhibitors, a 100 ns MD simulation was conducted. The study of parameters such as root mean square deviation (RMSD), root mean square fluctuations (RMSF), solvent accessible surface area (SASA), radius of gyration (Rg), and H bonds is valuable for analyzing structural changes and stability of the system. These parameters were examined for compounds 25 and 13, which were identified as the most active and inactive inhibitors in previous experimental work. RMSD and RMSF are key analytical parameters for demonstrating the stability and flexibility of a molecule. The RMSD analysis, in particular, is used to investigate the structural changes of a protein after interacting with a ligand. A decrease in the RMSD value of the protein following interaction with the ligand indicates increased stability. The RMSD diagram in Fig. 9 compares the RMSD for PIM1, 25-PIM1, and 13-PIM1 complexes. The average RMSD value for PIM1 was 0.31 ± 0.03 nm, while for complexes 25 and 13 it was calculated to be 0.25 ± 0.03 nm and 0.26 ± 0.03 nm, respectively. The research conducted by Beura et al. [44] suggests that a RMSD value below 3.0 nm indicates the stability of protein-ligand complexes in terms of their conformation. The RMSD diagram illustrates that after 80 ns of interaction, compound 25 forms a more stable complex with PIM1.

**Fig. 10:**
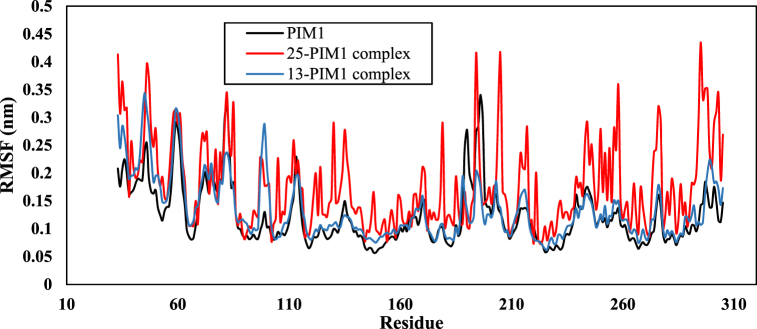
Chart of RMSF changes for PIM1, 25- PIM1 complex and 13- PIM1 complex.

The RMSF analysis compared the dynamics of individual residues from the protein backbone and estimated protein flexibility in complexes 25-PIM1 and 13-PIM1. This information is important for understanding the stability, rigidity, and compactness of the receptor. A high RMSF value suggests that the residue is flexible, whereas a low RMSF value suggests that the residue is stable [45]. For the 25-PIM1 complex, the minimum, maximum, and average RMSF fluctuation were 0.07, 0.43, and 0.17 ± 0.07 nm, respectively. For the 13-PIM1 complex, these values were 0.06, 0.35, and 0.14 ± 0.05 nm, indicating relatively low residual fluctuations (Fig. 10). Fig. 10 shows that most PIM1 interactions with ligands take place within residues 60 to 120, which is consistent with the results of the docking analysis.

**Fig. 11:**
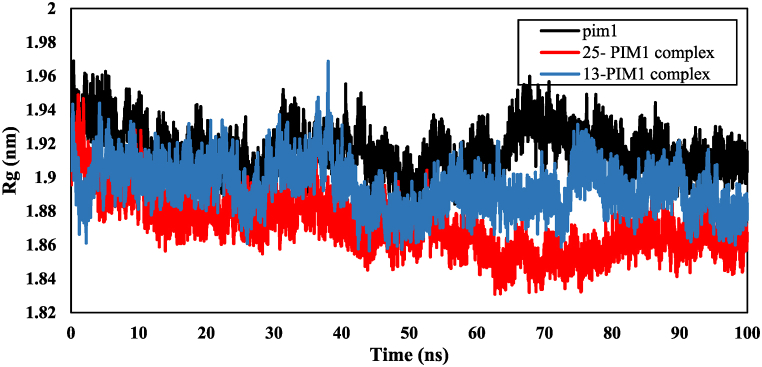
The radius of gyration (Rg) of the backbone atoms of PIM1, 25- PIM1complex and 13-PIM1 complex.

The gyration parameter's radius, which indicates the compactness of the protein structure, is used to assess the protein's stability. Fig. 11 displays the radius of gyration plot for PIM1, 25-PIM1, and 13-PIM1 complexes. The average radius of gyration for PIM1, 25-PIM1, and 13-PIM1 was 1.91 ± 0.013 nm, 1.87 ± 0.016 nm, and 1.89 ± 0.014 nm, respectively. This suggests that the radius of gyration of the complexes was less than that of the protein throughout the simulation. The decrease in the gyration radius of the complex compared to the protein indicates increased stability of the protein after binding to the ligand. Furthermore, the lower radius value for the 25-PIM1 complex compared to 13-PIM1 suggests a stronger interaction of compound 25 with the protein than compound 13.

**Fig. 12:**
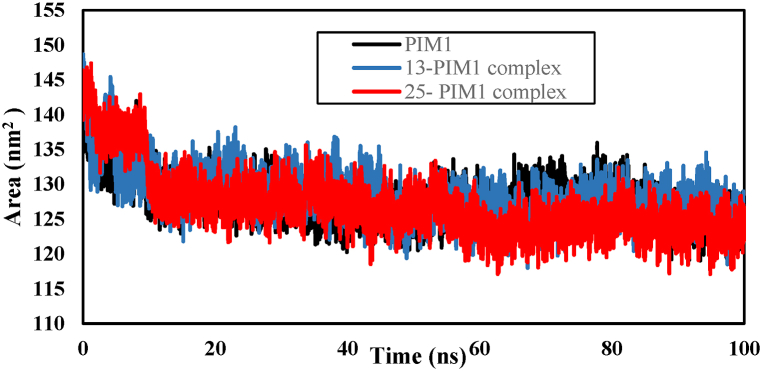
Solvent accessible surface area plot as a function of simulation time.

The solvent accessible surface area (SASA) is the surface area of a biological molecule that is available to the solvent. It is commonly used to study protein folding and stability. The decrease in SASA values of a protein suggests that the protein is becoming more compact and experiencing reduced exposure to solvents. The changes in SASA patterns over the 100 ns simulation time for the complexes were found to be similar to those of PIM1 kinase (Fig. 12). The average SASA values for PIM1 kinase, 25-PIM1, and 13-PIM1 complexes were 127.90, 127.08, and 128.40 ± 3.3 nm^2^, respectively. The SASA values remain relatively constant, suggesting that all complexes are compact. The SASA findings indicate that both compounds form stable complexes with PIM1.

**Fig. 13:**
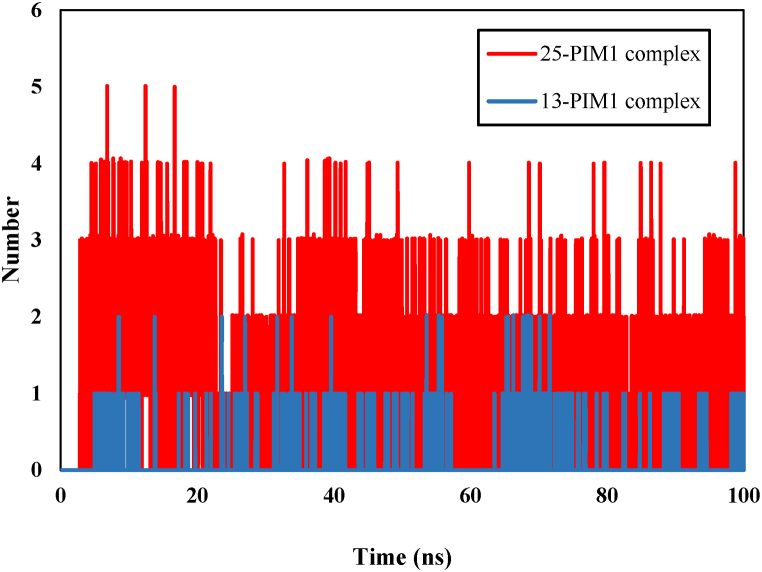
Hydrogen bonding analysis of 25-PIM1 and 13- PIM1 complexes.

During the 100 ns simulation, the investigation focused on the number of hydrogen bonds formed between compounds 25 and 13 with PIM1. The number of hydrogen bonds formed varied throughout the simulation. In the optimal scenario, compound 25 formed 5 bonds, while compound 13 formed 2 bonds with PIM1 (Fig. 13).

### 3.5 Design of novel compounds with higher inhibitory activity

According to the model created by QSAR and investigating the results of docking, interactions, and pharmacophore mapping study, 4 new compounds were designed as inhibitors of PIM1 with improved activity. The predicted structures and inhibitory efficiency and descriptor values for these compounds are shown in Table 9.

**Table 9:**
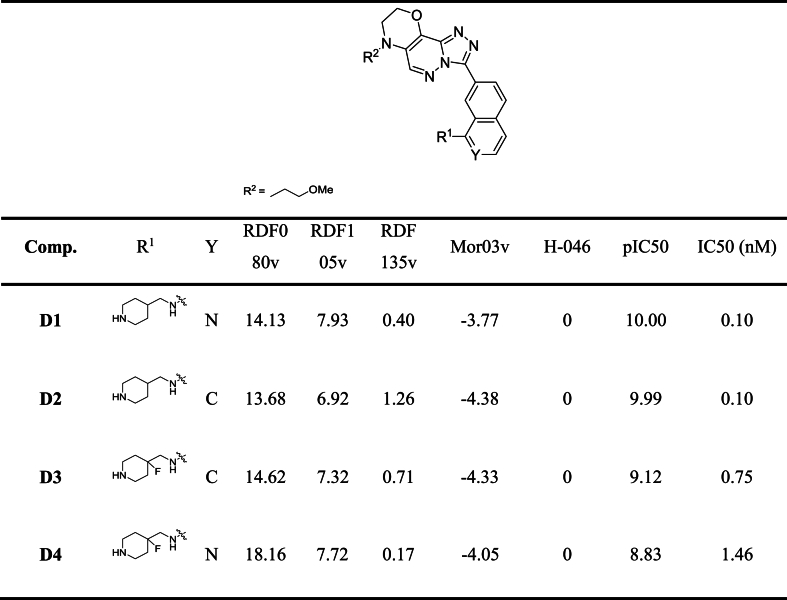
Structures and predicted inhibition efficiency values of new compounds based on the QSAR model.

**Fig. 14:**
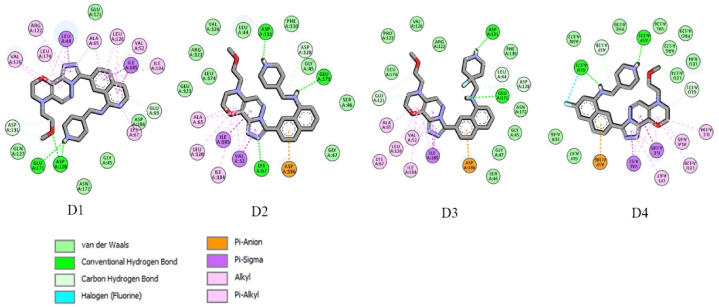
Docked poses of designed compounds D1, D2, D3 and D4.

The designed compounds demonstrate higher inhibition efficiency compared to the most active compounds in the data set. Molecular docking studies were conducted on these 4 compounds to confirm their inhibitory performance. The results indicate that the new compounds bind to PIM1 and exhibit an acceptable number of interactions (Fig. 14).

**Fig. 15:**
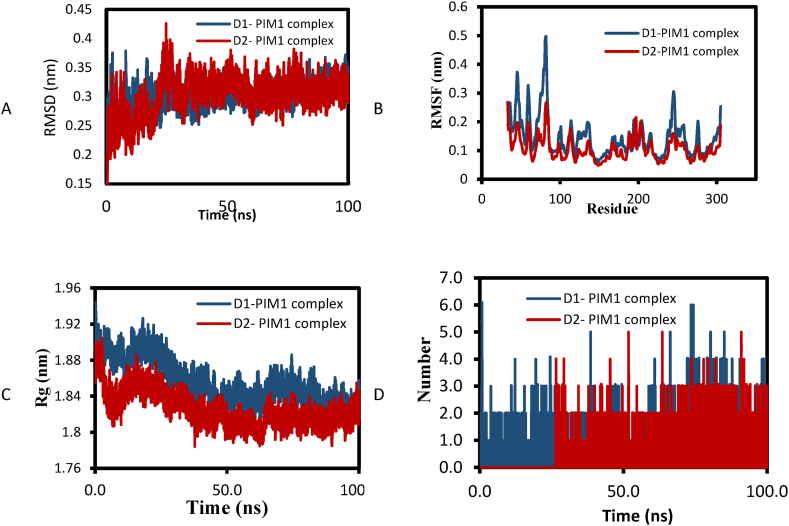
Results of MD simulation of D1- PIM1 and D2- PIM1 complexes: A) RMSD, B) RMSF, C) R_g_ and D) number of hydrogen bonds.

Two designed compounds with higher activity were subjected to molecular dynamics simulations. Fig. 15 illustrates the MD simulations findings for the two designed compounds interacting with PIM1 during a 100 ns simulation. The RMSD results show that the D1-PIM1 and D2- PIM1 complexes experienced significant stability with an average of 0.3 nm (Fig. 15A). The RMSF results (Fig. 15B) show that the studied ligands have some affinity for stable regions of residues. Fig. 15C displays the results of Rg over the course of a 100 ns simulation. The minimal variation in Rg observed in all complexes indicates a significant interaction between the compounds and PIM1.The analysis of H-bonds in Fig. 15D shows that D1 can form 1 to 6 H-bonds, while D2 can form 1 to 5 H-bonds. The ligands remain stable because of the formation of various hydrogen bonds, indicating their significant interaction with PIM1 during the 100 ns simulation.

**Fig. 16:**
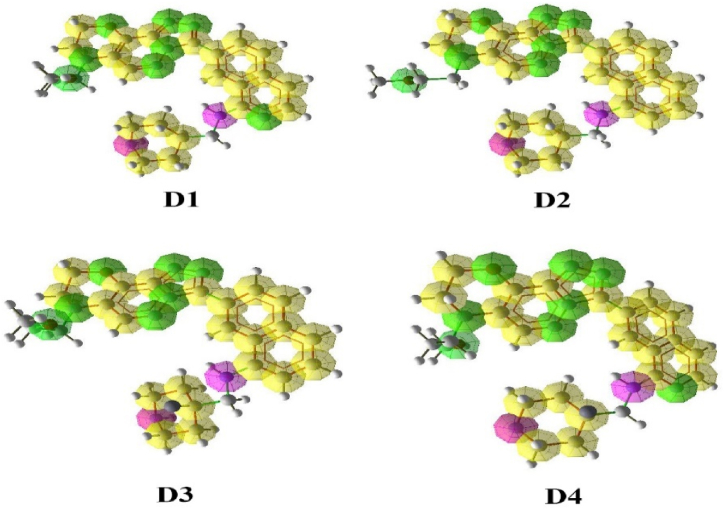
Pharmacophore Mapping of designed compounds D1, D2, D3 and D4. Here, green color-hydrogen bond acceptor, purple color-hydrogen bond donor and yellow color-aromatic.

Also, pharmacophore mapping was performed for compounds D1, D2, D3 and D4 (Fig. 16). The increase of hydrogen acceptors in the pharmacophore map of the designed compounds indicates the possibility of increasing the interaction between ligands and PIM-1 kinase. The pharmacophore mapping results confirmed the docking results.

In the final approval of these compounds, the ADMET method [46] was used to identify potential new medicinal compounds. ADMET is a modern method used to identify molecules with potential as drug candidates, focusing on their effectiveness and safety. Investigating the absorption, distribution, metabolism, excretion, and toxicity (ADMET) of medicinal substances is crucial in the discovery and development of drugs. Therefore, the designed molecules were evaluated for ADMET and compliance with the rule of 5. The Swiss ADME server (http://www.swissadme.ch) [47] and ADMETlab 2.0 (ADMETlab 2.0 (scbdd.com) [48] were used to assess the drug-likeness properties, and the results are shown in Table 10.

**Table 10:** Drug-likeness properties and in silico predicted ADMET properties for the designed compounds.

Comp.	25	D1	D2	D3	D4
Molecular weight (g/mol)	430.51	474.56	473.57	491.56	492.55
Num. of rotatable bonds	4	7	7	7	7
Num. of H-bond donors	2	2	2	2	2
Num. of H-bond acceptors	6	7	6	7	8
TPSA (Topological Polar surface area)	92.5	101.73	88.84	88.84	101.73
Molar Refractivity	130.98	141.68	143.89	143.98	141.77
XLOGP	2.08	1.93	2.67	2.64	1.91
WLOGP	1.63	1.64	2.25	2.76	2.16
MLOGP	1.82	1.47	2.03	2.14	1.57
Lipinski's violations	Yes	Yes	Yes	Yes	Yes
Veber Violations	Yes	Yes	Yes	Yes	Yes
Egan Violation	Yes	Yes	Yes	Yes	Yes
Gastrointestinal absorption	High	High	High	High	High
BBB permeant	No	Yes	No	Yes	Yes
VDs	1.75	1.68	1.76	1.80	1.75
Substrate of P-gp	Yes	Yes	Yes	Yes	Yes
CYP1A2 inhibitor	Yes	No	No	No	No
CYP2C19 inhibitor	No	Yes	Yes	Yes	Yes
CYP2C9 inhibitor	Yes	Yes	Yes	Yes	Yes
CYP2D6 inhibitor	Yes	Yes	Yes	Yes	Yes
CYP3A4 inhibitor	Yes	Yes	Yes	Yes	Yes
CL	7.84	8.79	9.15	7.99	7.36
AMES Toxicity	No	No	No	No	No
Rat Oral Acute Toxicity	No	No	No	No	No

According to Lipinski's rule of 5, a molecule with characteristics such as a molecular mass lower than 500 Da, less than 5 hydrogen donors, less than 10 hydrogen acceptors, and a calculated Log P (CLogP) less than 5 (or MlogP <4.15) can be well absorbed through food. The designed compounds in Table 9 exhibit these characteristics and also comply with the Veber, Egan, and Muegge rules. Therefore, they can be considered as potential new medicinal compounds.

Pharmacokinetic data evaluation indicates that the designed compounds have high intestinal absorption rates and oral bioavailability. The volume distribution (VDs) in the range of 0.04–20 L/kg suggests a high distribution index for the compounds. Analysis of the compounds' permeability into the blood-brain barrier (BBB) shows that compounds D1, D3, and D4 can permeate through the BBB, while compound D2 cannot. Enzymatic metabolism involves the chemical transformation of drugs in the human body, which is essential for maintaining the stability of drugs in the body. The liver contains the main enzymes responsible for drug metabolism, including cytochrome P450 enzymes (CYP1A2, CYP3A4, CYP2C19, CYP2D6, and CYP2C9), which are responsible for metabolizing over 90 % of drugs. Inhibiting these enzymes can lead to an increase in the concentration of the active drug in the body [37]. Additionally, a clearance (CL) higher than 5 ml/min/Kg indicates that the designed compounds are stable and can reach therapeutic goals before excretion. The AMES test and Rat Oral Acute Toxicity assessments in Table 10 demonstrate that none of the designed compounds exhibit toxicity.

## 4 Conclusion

In the current study, we investigated the effectiveness of 30 triazolo [4, 3-b] pyridazin-3-yl-quinoline derivatives in inhibiting PIM1 kinase aggregation. This was done through a QSAR study, molecular docking, and MD simulations. The QSAR method involved linear modeling using the MLR method, incorporating 5 descriptors: RDF080v, RDF105v, RDF135v, Mor03v, and H046. Among these five descriptors, Mor03v indicated the greatest positive impact on the activity. Subsequently, nonlinear SVR modeling was conducted that a model with R^2^ = 0.97 was created. The validity of the obtained models was confirmed using the Y-Randomization evaluation method. Molecular docking studies were performed to examine the binding modes of compounds at the active site of PIM1 kinase, revealing key residues such as ASP128, GLU171, LYS67, and GLY50 at the PIM1 binding site for interaction with the inhibitors. According to the molecular docking results, it was observed that compounds 25 and 26 have the highest number of hydrogen bonds with the receptor and have the highest binding energy. MD simulation was carried out on compound 25, exhibiting the highest inhibition efficiency, and compound 13, showing the lowest inhibition efficiency, confirming that compound 25 could form a stable complex with PIM1.

Based on the QSAR modeling and the ligand-PIM1 interactions identified through molecular docking, four new inhibitors with improved inhibition efficiency were successfully designed. The activity of newly designed compounds was calculated using the model. The interactions of these compounds with the receptor were also investigated by molecular docking and MD simulation. Finally, these new compounds showed promising drug-like properties, including high oral bioavailability, minimal toxicity, and efficient cell membrane permeability as predicted by ADMET analysis. These characteristics suggest significant potential for further experimental research to explore their pharmacological effects in both in vitro and in vivo.

Ultimately, the findings from this study provide a strong foundation for the development of new PIM1 kinase inhibitors. The promising results from QSAR modeling, molecular docking, and MD simulations offer valuable insights into the design of effective inhibitors, paving the way for future experimental validation and potential therapeutic applications.

## Data availability statement

Data will be made available on request.

## CRediT authorship contribution statement

**Fereshteh Golestanifar:** Writing – original draft, Validation, Software, Formal analysis. **Zahra Garkani-Nejad:** Writing – review & editing, Supervision, Investigation.

## Declaration of competing interest

The authors declare that they have no known competing financial interests or personal relationships that could have appeared to influence the work reported in this paper.
